# Analysis of Paramyxovirus Transcription and Replication by High-Throughput Sequencing

**DOI:** 10.1128/JVI.00571-19

**Published:** 2019-08-13

**Authors:** Elizabeth B. Wignall-Fleming, David J. Hughes, Sreenu Vattipally, Sejal Modha, Steve Goodbourn, Andrew J. Davison, Richard E. Randall

**Affiliations:** a School of Biology, Centre for Biomolecular Sciences, University of St. Andrews, St. Andrews, Fife, United Kingdom; b MRC-University of Glasgow Centre for Virus Research, Glasgow, United Kingdom; c Division of Basic Medical Sciences, St. George's, University of London, London, United Kingdom; University of Kentucky College of Medicine

**Keywords:** high-throughput sequencing, paramyxovirus, replication, transcription

## Abstract

We have developed a high-throughput sequencing (HTS) workflow for investigating paramyxovirus transcription and replication. We show that sequencing of oligo(dT)-selected polyadenylated mRNAs, without considering the orientation of the RNAs from which they had been generated, cannot accurately be used to analyze the abundance of viral mRNAs because genomic RNA copurifies with the viral mRNAs. The best method is directional sequencing of infected cell RNA that has physically been depleted of ribosomal and mitochondrial RNA followed by bioinformatic steps to differentiate data originating from genomes from viral mRNAs and antigenomes. This approach has the advantage that the abundance of viral mRNA (and antigenomes) and genomes can be analyzed and quantified from the same data. We investigated the kinetics of viral transcription and replication during infection of A549 cells with parainfluenza virus type 2 (PIV2), PIV3, PIV5, or mumps virus and determined the abundances of individual viral mRNAs and readthrough mRNAs. We found that the mRNA abundance gradients differed significantly between all four viruses but that for each virus the pattern remained relatively stable throughout infection. We suggest that rapid degradation of non-poly(A) mRNAs may be primarily responsible for the shape of the mRNA abundance gradient in parainfluenza virus 3, whereas a combination of this factor and disengagement of RNA polymerase at intergenic sequences, particularly those at the NP:P and P:M gene boundaries, may be responsible in the other viruses.

**IMPORTANCE** High-throughput sequencing (HTS) of virus-infected cells can be used to study in great detail the patterns of virus transcription and replication. For paramyxoviruses, and by analogy for all other negative-strand RNA viruses, we show that directional sequencing must be used to distinguish between genomic RNA and mRNA/antigenomic RNA because significant amounts of genomic RNA copurify with poly(A)-selected mRNA. We found that the best method is directional sequencing of total cell RNA, after the physical removal of rRNA (and mitochondrial RNA), because quantitative information on the abundance of both genomic RNA and mRNA/antigenomes can be simultaneously derived. Using this approach, we revealed new details of the kinetics of virus transcription and replication for parainfluenza virus (PIV) type 2, PIV3, PIV5, and mumps virus, as well as on the relative abundance of the individual viral mRNAs.

## Abstract (precis)

High-throughput sequencing (HTS) of virus-infected cells can be used to study in great detail the patterns of virus transcription and replication. For paramyxoviruses, and by analogy for all other negative-strand RNA viruses, we show that directional sequencing must be used to distinguish between genomic RNA and mRNA/antigenomic RNA because significant amounts of genomic RNA copurify with poly(A)-selected mRNA. We found that the best method is directional sequencing of total cell RNA, after the physical removal of rRNA (and mitochondrial RNA), because quantitative information on the abundance of both genomic RNA and mRNA/antigenomes can be simultaneously derived. Using this approach, we revealed new details of the kinetics of virus transcription and replication for parainfluenza virus (PIV) type 2, PIV3, PIV5, and mumps virus, as well as on the relative abundance of the individual viral mRNAs.

## INTRODUCTION

The family *Paramyxoviridae* belongs to the order *Mononegavirales* and is populated by a large number of vertebrate viruses, some of which cause diseases in humans, including mumps, measles, and respiratory infections (https://talk.ictvonline.org/taxonomy/). Parainfluenza virus 2 (PIV2), parainfluenza virus 5 (PIV5) and mumps virus (MuV) are members of the species *Human orthorubulavirus 2*, *Mammalian orthorubulavirus 5*, and *Mumps orthorubulavirus*, respectively, in genus *Orthorubulavirus* of subfamily *Rubulavirinae*. Parainfluenza virus 3 (PIV3) is a member of the species *Human respirovirus 3* in genus *Respirovirus* of subfamily *Orthoparamyxovirinae*; measles virus is a member of the species *Measles morbillivirus* in genus *Morbillivirus* of the same subfamily.

**FIG 1:**
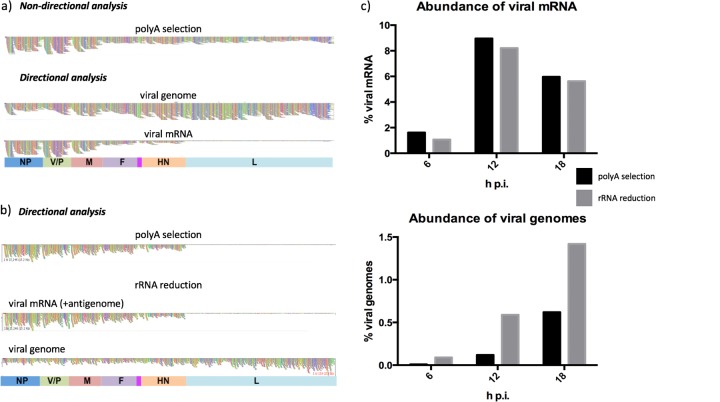
Optimization of a workflow to study PIV5-W3 transcription and replication by nondirectional analysis of HTS data followed by directional analysis to distinguish mRNA/antigenome reads from genome reads. In panels a and b, colored boxes indicate approximate gene positions and contain the names of the genes. The individual colored vertical bars represent the coverage depth (number of reads) at each nucleotide in the reference sequence. (a) BWA alignments of the PIV5-W3 transcriptome in HSFs at 18 h p.i. analyzed using poly(A)-selected RNA and visualized in Tablet. (b and c) Comparison of mRNA/antigenome and genome RNA abundance relative to total RNA after poly(A) selection or rRNA reduction of total cell RNA. RNA was extracted from PIV5-W3-infected A549 cells at 6, 12, and 18 h p.i., and the reads were subjected to directional analysis. (b) BWA alignments for mRNA/antigenome and genome reads at 18 h p.i. visualized in Tablet. (c) Abundance of mRNA/antigenome and genome reads at 6, 12, and 18 h p.i.

Paramyxoviruses possess single-stranded, nonsegmented, negative-sense RNA genomes that are typically 15,000 to 19,000 nucleotides (nt) in size. The genomes of different paramyxoviruses encode comparable, but not identical, cohorts of genes that exhibit largely analogous functions (see Fig. 1 for the layout in PIV5). The 3′ end of the genome contains an extracistronic region of 55 to 70 nt, which makes up the leader (Le) region and contains the Le promoter elements required for generation of viral mRNAs and antigenomes. The first promoter element is a conserved string of approximately 13 nucleotides at the 3′ end of the genome; the second element is tandem repeats in the untranslated region of the NP gene. These repeats must be in the correct position in relation to their encapsidating NP monomer known as hexamer phase. The 5′ end of the genome contains an extracistronic region of 21 to 161 nt that is known as the trailer (Tr) region. Viral mRNAs are transcribed by a stop-start process that is directed by *cis*-acting elements in the genome. These elements include the gene start (GS) and gene end (GE) sites that flank the individual genes. Immediately downstream of the GE site is a poly(U) tract of variable length, which forms the site of mRNA polyadenylation. Between each pair of genes there is an additional *cis*-acting element known as the intergenic (IG) region, which consists of a short sequence (1 to 56 nt) that is not generally transcribed into mRNA. IG regions vary in sequence and length among paramyxovirus genera. Respiroviruses and morbilliviruses have IG regions that are conserved in length and sequence within the genome, whereas rubulaviruses possess IG regions that vary in length and sequence throughout the genome (for reviews of the molecular biology of paramyxoviruses, see references 1 and 2).

The processes of transcription and replication are similar in members of the order *Mononegavirales* (3). Upon entry of the virus into the cell, primary transcription of genomes to generate mRNAs is initiated by the virion-associated viral RNA-dependent RNA polymerase complex (vRdRP), which, in the case of paramyxoviruses, consists of the large protein (L) and the phosphoprotein (P). Only after sufficient amounts of soluble NP (NP^0^), which is kept soluble by its interaction with the N-terminal common domain of P and V (4–7), have been produced does virus replication begin, as NP^0^ is required for encapsidation of newly synthesized genomes and antigenomes (8, 9). The new viral genomes then act as templates for secondary transcription and replication.

During transcription, vRdRP attaches to the Le promoter and scans along the genome until it reaches the first GS site, where it initiates transcription of the NP gene. The GS site is thought to contain the signal for vRdRP to carry out capping and cap methylation (10–12). After transcription of the NP gene, polyadenylation occurs by stuttering of vRdRP in the 4 to 7 U residues following the GE site. An mRNA that is 5′ capped and methylated and 3′ polyadenylated is then released. The generally accepted model is that vRdRP then either disengages from the genome at the GE or traverses the IG region to reinitiate transcription at the GS site of the next gene. If vRdRP disengages from the genome, it can participate in further transcription only by reinitiating transcription at the Le promoter. This mechanism, known as stop-start transcription, produces a transcriptional gradient, with greater quantities of mRNA being produced from genes nearer the 3′ end of the genome (13–16). With time postinfection (p.i.) not only will the rate of production of individual viral mRNAs determine their relative abundance but also their relative rate of degradation. Throughout this article we therefore refer to mRNA abundance gradients rather than transcriptional gradients. During transcription, vRdRP sometimes fails to terminate transcription at the GE site. When this happens, vRdRP transcribes the IG region and downstream gene(s), producing a polycistronic or readthrough mRNA. A shared characteristic of paramyxovirus transcription is a higher rate of readthrough at the M:F boundary. The mechanism that directs the rates of readthrough at the gene junctions is unclear. A series of papers by Rassa et al. (17–19) identified the GE site and the first nucleotide of the IG region to be important in generating a greater abundance of M:F readthrough mRNA and suggested that these elements may work in tandem to direct the vRdRP. Unlike for vesicular stomatis virus (VSV) of the order *Mononegavirales* from the *Rhabdoviridae* family, which is thought to have similar transcription and replication mechanisms, altering the length of the IG region did not effect the frequency of M:F mRNA readthroughs. Furthermore, these papers suggested that the U tract and IG region might act as a spacer between the GE and GS sites and play an important role in transcriptional initiation at the next gene (19).

Paramyxoviruses share the common feature of allowing multiple mRNAs to be transcribed from the P/V gene by a process known as RNA editing. This is where additional G residues are inserted at a specific position in a proportion of mRNAs, facilitating a translational frameshift. RNA editing occurs by slippage of vRdRP within a short poly(G) tract, in a manner similar to that occurring during polyadenylation (20, 21). In orthorubulaviruses, the V/P gene produces three transcripts: V, which is a faithful copy of the gene; P, which is generated by insertion of two G residues at the RNA editing site of the P transcript; and I, which is produced by insertion of a single G residue. As a result, the V, P, and I proteins share the same N-terminal sequence but differ in their C-terminal sequences. In respiroviruses, P is a faithful copy of the gene, and mRNAs encoding D and V are generated by insertion of one or two G residues, respectively. A number of paramyxoviruses also produce one or more C proteins from an additional open reading frame (ORF) present upstream of the RNA editing site that generates the P, D, and V mRNAs.

During replication the vRdRP attaches to the Le promoter and transcribes the entire genome, ignoring all GS and GE sites. This produces a full-length, faithful, positive-sense copy of the genome known as the antigenome, which acts as a template for production of viral genomes. The complement of the Tr region, the 3′ end of the antigenome, contains the antigenome promoter (TrC) elements required for RdRp polymerase recognition and initiation of the production of *de novo* genomes. The newly synthesized genomes and antigenomes are concurrently encapsidated by NP^0^ to form the nucleocapsid structure. It is thought that concurrent replication and encapsidation allow vRdRP to ignore GS and GE sites (22, 23).

Despite this general understanding of the general patterns of paramyxovirus transcription and replication, detailed descriptions are lacking for most individual paramyxoviruses. In the present study, we exploited high-throughput sequencing (HTS) to analyze simultaneously the kinetics of transcription and replication of several paramyxoviruses, thus potentially also shedding light on these processes in all members of the order *Mononegavirales*.

## RESULTS

### Transcription and replication in PIV5.

In preliminary studies, untransformed human skin fibroblasts (HSFs) (that had undergone only limited passage in tissue culture cells) were infected with PIV5-W3 at a multiplicity of infection (MOI) of 50 PFU/cell. RNA was extracted at 18 h postinfection (p.i.), and mRNA was isolated by poly(A) selection prior to HTS on the MiSeq platform. The resulting R1 and R2 files contained a total of 6,523,498 reads, which were trimmed and mapped to the PIV5-W3 genome sequence without considering the orientation of the RNAs from which they had been generated. Viral reads accounted for 4.7% of the total. Coverage depth of the NP and V/P genes was greater than that of other genes, reflecting the anticipated mRNA abundance gradient (Fig. 1a, top). However, downstream genes, including the L gene, displayed approximately equivalent coverage depths, implying that the gradient did not extend to these genes. An alternative explanation is that the poly(A)-selected RNA preparation contained significant amounts of genomes and antigenomes. To determine whether this was the case, the orientation of the original RNAs (viral genomes are negative sense and viral mRNAs/antigenomes are positive sense) was considered by mapping the genome and mRNA/antigenome reads independently to the PIV5-W3 sequence (Fig. 1a, middle and bottom). Although mRNA/antigenome reads accounted for 2.2% of total reads, genome reads accounted for more (2.5%), showing that significant amounts of genome RNA were present in the poly(A)-selected RNA preparation. Alignment of mRNA/antigenome reads revealed a clear mRNA abundance gradient, with greater coverage depth in genes at the 3′ end of the genome (NP and V/P) and significantly less coverage depth in the L gene at the 5′ end (Fig. 1a, bottom). Although it is not possible to distinguish reads generated from mRNAs from those generated from antigenomes by directional sequencing, the proportion of antigenome reads cannot exceed that of the L gene extended over the whole genome (2.6% of mRNA/antigenome reads overall). Finally, by calculating the average coverage depth of reads at positions 45 to 54 in the Le region (which is not included in mRNAs), it was estimated that antigenomes contributed only 0.05% of mRNA/antigenome reads.

Although viral genomes copurified with mRNA during poly(A) selection most likely due to hybridization of cRNA during RNA extraction, the number of viral genomes in infected cells could not be quantified because the efficiency of selection was not known. Therefore, we investigated whether directional sequencing following depletion of rRNA, rather than poly(A) selection, could achieve the quantification of both genome and mRNA/antigenome RNA from the same data set. A549 cells were infected with PIV5-W3 at an MOI of 10 PFU/cell. RNA was extracted at 6, 12, and 18 h p.i. and subjected to rRNA reduction or poly(A) selection prior to HTS on the MiSeq platform. The resulting R1 and R2 files were processed into genome and mRNA/antigenome files and mapped to the PIV5-W3 sequence. Since neither poly(A) selection nor depletion of rRNA was capable of completely removing rRNA from the samples, and also did not remove mitochondrial RNA, residual rRNA and mitochondrial reads were removed bioinformatically from this point (Table 1). The abundance of mitochondrial RNA reads was particularly apparent in the rRNA reduction approach and indicated that a physical method that reduces both rRNA and mitochondrial RNA prior to sequencing may, under certain circumstances, be the most appropriate method to use.

**TABLE 1:** Percentages of PIV5 strain W3 viral mRNA reads compared to total reads before and after rRNA and mitochondrial RNA reads had been bioinformatically removed from the data obtained using poly(A) selection or rRNA reduction library preparation

Procedure and no. of hours p.i.	% of PIV5 strain W3 mRNA reads			
	Before reads removed, mRNA	Reads in data sets		After reads removed, mRNA
		rRNA	Mitochondrial	
Poly(A) selection				
6	1.5	1.6	8.5	1.6
12	8.2	1.6	6.1	8.9
18	5.4	3.1	7.3	5.9
rRNA reduction				
6	1.0	0.4	3.8	1.1
12	7.2	0.2	11.8	8.2
18	4.8	1.9	13.2	5.6

No significant differences were observed between poly(A) selection and rRNA reduction in terms of either relative mRNA abundance or the shape of the mRNA abundance gradient (Fig. 1b and c; a quantitative description of the mRNA abundance gradient is provided below). For example, the observation that the mRNA profile at 12 h p.i. for poly(A)-selected RNA was essentially indistinguishable from that for rRNA-depleted RNA (Fig. 1b) indicated that directional sequencing of total infected cell RNA, incorporating both physical and bioinformatic removal of rRNA reads (and bioinformatic removal of mitochondrial RNA reads), can be used to investigate the mRNA abundance gradient of PIV5 and thus potentially of all negative-strand RNA viruses. The advantage of rRNA reduction over poly(A) selection is that it facilitates quantification of the abundance of both genome and mRNA/antigenome reads in the same data set (Fig. 1c). Indeed, the amount of viral genomes present in poly(A)-selected RNA proved to be significantly smaller than that in rRNA-reduced RNA, presumably because not all genomes copurified with mRNA during poly(A) selection. The abundance of genome reads determined from rRNA reduction data increased gradually between 6 and 18 h p.i. from 0.09 to 1.42% of total reads. Interestingly, a gradient of genome reads from the Tr region was visualized at 12 h p.i. (Fig. 1b), perhaps because incomplete replicating genomic RNA had been sequenced. Additionally, the proportions of antigenomes at 6, 12, and 18 h p.i. were estimated from coverage at positions 45 to 54 that was extended to the whole genome and were estimated as 0.07, 0.21, and 0.16%, respectively, of total reads. In addition, to quantify the amount of genomic RNA present, sequencing of total infected cell RNA also facilitates the detection and quantification of defective interfering genomes (24).

The analysis described above involved physical reduction of rRNA. However, a significant proportion of reads originated from mitochondrial RNA (Table 1). All subsequent experiments were conducted using physical reduction of rRNA and mitochondrial RNA followed by bioinformatic removal of residual rRNA and mitochondrial RNA reads. In addition, all subsequent samples were sequenced using the NextSeq, rather than MiSeq, platform, in order to generate more reads. Following sequencing, the bioinformatic pipeline described above was key to the analysis, as it allowed genome and mRNA/antigenome reads to be distinguished from each other.

### Analysis of transcription and replication in other paramyxoviruses.

**FIG 2:**
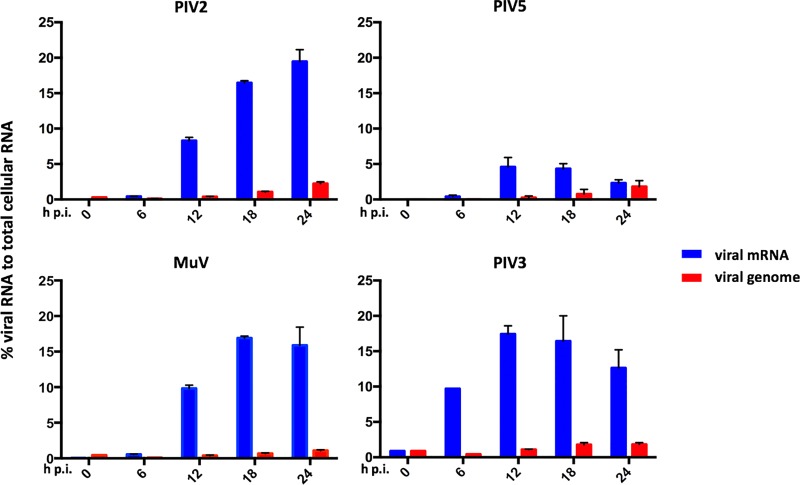
Kinetic analysis of PIV2-Co, PIV3-Wash, PIV5-W3, and MuV-Enders transcription and replication. The relative abundances of mRNA and genome reads were compared to the number of total reads at various times p.i. A549 cells were infected at an MOI of 10 to 20 PFU per cell, and total RNA was isolated at various times p.i. Following physical removal of rRNA and mitochondrial RNA, the samples were subjected to library preparation, sequencing, and directional analysis, followed by bioinformatical removal of residual rRNA and mitochondrial reads. The bars show SD values based on three experiments.

The workflow described above was used to investigate and compare the rates of viral mRNA and genome accumulation of PIV2-Co, PIV3-Wash, PIV5-W3, and MuV-Enders. Triplicate cultures of A549 cells were infected with the individual viruses at an MOI of 10 to 20 PFU/cell. Total infected cell RNA was isolated at 0, 6, 12, 18, and 24 h p.i. and processed for sequencing and subsequent bioinformatic analysis (Fig. 2). Since we had estimated that antigenome reads form a very small proportion of mRNA/antigenome reads, we abbreviated below “mRNA/antigenome reads” to just “mRNA reads” where appropriate.

PIV3-Wash exhibited significantly faster transcriptional kinetics than the other viruses, with mRNA contributing approximately 10% of total RNA at 6 h p.i. and reaching maximal levels (approximately 18%) by 12 h p.i. In contrast, the levels of PIV2-Co, PIV5-W3, and MuV-Enders transcripts were <2% of total RNA at 6 h p.i. The greatest increase in the rate of viral transcription for PIV2-Co, PIV5-W3, and MuV-Enders was observed between 6 and 12 h p.i. However, the pattern of PIV5-W3 transcription differed significantly at later times from that of MuV-Enders and PIV2-Co, with mRNA levels peaking at 16 to 19% of total RNA at 18 and 24 h p.i., respectively. In contrast, the levels of PIV5-W3 mRNA peaked between 12 and 18 h p.i., contributing 4 to 5% of total RNA, after which the abundance decreased to 2 to 3% by 24 h p.i. This reflects an almost 4-fold difference in peak mRNA abundance between PIV5-W3 and PIV2-Co and MuV-Enders (discussed further below). Despite differences in the kinetics of transcription and relative abundances of the PIV2-Co, PIV5-W3, and MuV-Enders mRNAs, the abundance of viral genomes gradually increased for all three viruses between 6 and 24 h p.i. from approximately 0.03 to 1 to 2% of total RNA. As would be expected from the higher rate of transcription in PIV3-Wash, replication was also slightly faster, with a significant increase in viral genome numbers being observed between 6 and 12 h p.i., reaching maximal levels by 18 h p.i.

### Viral mRNA abundance gradients.

**FIG 3:**
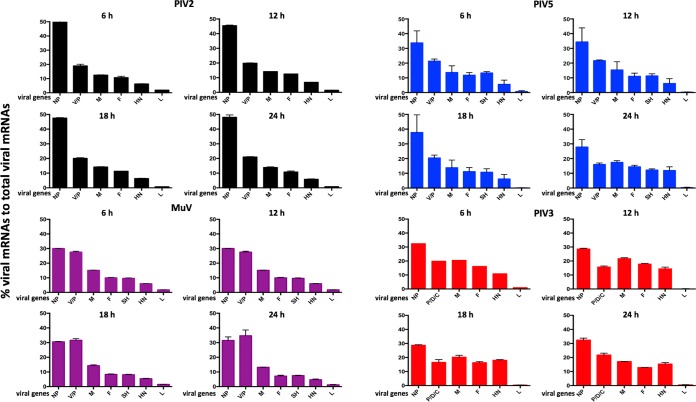
Comparison of the mRNA abundance gradients of PIV2-Co, PIV3-Wash, PIV5-W3, and MuV-Enders with time p.i. The RNA samples described for Fig. 2 were subjected to bioinformatic analysis to determine the percent contribution of individual viral mRNAs to the total viral mRNA population.

The viral mRNA abundance gradients were analyzed in the above-named samples by determining the relative abundance of individual viral mRNAs using values for fragments per kilobase of transcript per million mapped reads (FPKM), which take into account gene length in order to allow the relative amounts of mRNA transcribed from individual genes to be compared. These values were then used to determine the percent contribution of each viral mRNA to the total (Fig. 3).

There were significant differences between the transcriptional profiles of the four viruses. For PIV2-Co and PIV5-W3, the NP mRNAs were clearly the most abundant, contributing >45% of total mRNA in the case of PIV2-Co. There was then a relatively steep reduction in the abundance of the V/P mRNAs and then a more gradual decline until the HN mRNA, followed by a sharp decline in the abundance of L mRNA, particularly for PIV3 and PIV5. In contrast, the relative levels of the NP and V/P mRNAs were similar for MuV-Enders, with a relatively steep reduction to the M mRNA. For PIV3-Wash, there was a more gradual decline until the sharp decrease in the abundance of the L mRNA. Unexpectedly, although not open to meaningful statistical analysis, the relative abundance of the PIV3-Wash P/V/D mRNAs in most samples appeared to be slightly less than that of the M mRNA. Assuming that there is no internal entry site for vRdRP, this may reflect differences in mRNA stability. This may also explain the slight apparent differences observed in the mRNA abundance gradients for each virus at different time points. However, the fact that the transcriptional profiles at later time points were similar to those at 6 h p.i., a time when the relative stability of different viral mRNAs is unlike to significantly affect the mRNA abundance gradients, suggests that there is no significant temporal control of the levels of viral transcription of individual genes.

### RNA editing.

**FIG 4:**
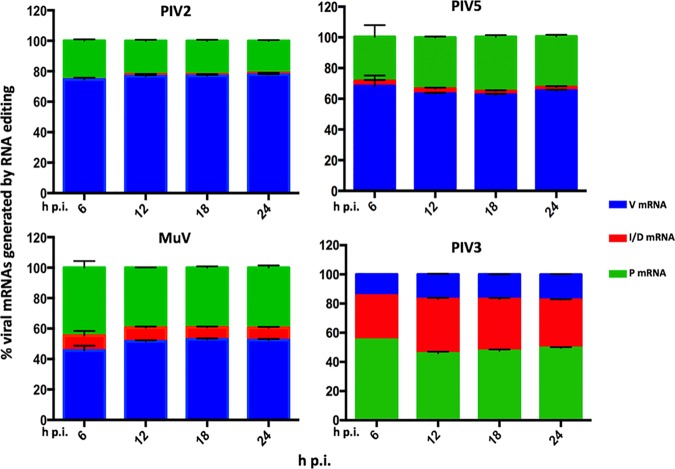
Analysis of RNA editing. Shown are relative abundances of the P, V, and I mRNAs for PIV2-Co, PIV5-W3, and MuV-Enders (orthorubulaviruses) and the P, V, and D mRNAs for PIV3-Wash (respiroviruses) in the RNA samples described for Fig. 2. The number of reads generated from the RNA editing site was calculated using a 10-nt search string immediately upstream and downstream of the site. The number of inserted G residues in the reads overlapping the RNA editing site that generated the V, P, and I mRNA transcripts was calculated, 0 and 0 + 3 G inserts (V or P for orthorubulaviruses and respiroviruses, respectively), 2 and 2 + 3 G inserts (P or D for orthorubulaviruses and respiroviruses, respectively), and 1 and 1 + 3 G inserts (I or V for orthorubulaviruses or respiroviruses, respectively). The bars show SD values based on three independent experiments.

The distribution of additional G residues inserted at the editing site into the relevant mRNAs is shown in Table 2. The editing profiles of PIV2-Co and PIV5-W3 were similar to each other (Fig. 4). The ratios of V (unedited) to P (edited) mRNA were approximately 2:1 and 3:1, respectively. Together these mRNAs accounted for approximately 98% of reads overlapping the editing site in PIV2-Co and 94% in PIV5-W3, with the I (edited) mRNA accounting for <2% of reads. Edited mRNAs with >2 G inserted residues contributed <1% and <3% of the total V/P/I mRNA population for PIV2-Co and PIV5-W3, respectively (Table 2). In contrast to the other orthorubulaviruses, the V (unedited) mRNA for MuV-Enders was only slightly more abundant than P (edited) mRNA, and I (edited) mRNA was 5% of the total V/P/I mRNA population (Fig. 4). Furthermore, editing was less precise for MuV-Enders than PIV2-Co and PIV5-W3, in that the number of mRNAs with 3 and 4 inserted G residues amounted to approximately 8 to 9% of reads overlapping the editing site (Table 2). For PIV3-Wash, the P, D, and V mRNAs were present at a ratio of approximately 3:2:1 (Fig. 4). This result is in contrast to that observed by Kolakofsky et al. (25), who reported that PIV3 inserts 1 to 6 G residues at the editing site with equal frequency.

**TABLE 2:** Mean percentages of reads containing additional inserted G residues compared with total number of reads overlapping the V/P RNA editing site

Virus	No. of hours p.i.	% of reads containing indicated no. of additional inserted G residues							
		0	1	2	3	4	5	6	7
PIV2-Co	6	74	0	25	1	0.0	0.0	0.0	0.0
	12	76	1	22	1	0.1	0.0	0.0	0.0
	18	76	1	22	1	0.1	0.0	0.0	0.0
	24	77	1	21	1	0.1	0.0	0.0	0.0
PIV5-W3	6	64	2	29	2	1	2	0.0	0.0
	12	60	2	33	2	2	1	0.1	0.0
	18	59	1	35	3	1	1	0.1	0.0
	24	62	1	33	2	1	0	0.1	0.0
MuV Enders	6	41	6	44	5	4	1	0.2	0.1
	12	46	5	39	6	4	1	0.0	0.0
	18	47	4	38	6	4	1	0.1	0.0
	24	48	5	39	5	3	0.4	0.1	0.0
PIV3-Wash	6	47	24	8	7	6	7	0.6	0.2
	12	39	27	10	7	10	7	0.5	0.3
	18	40	26	10	7	10	7	0.6	0.2
	24	41	24	10	8	9	8	0.8	0.4

### Readthrough mRNAs.

**FIG 5:**
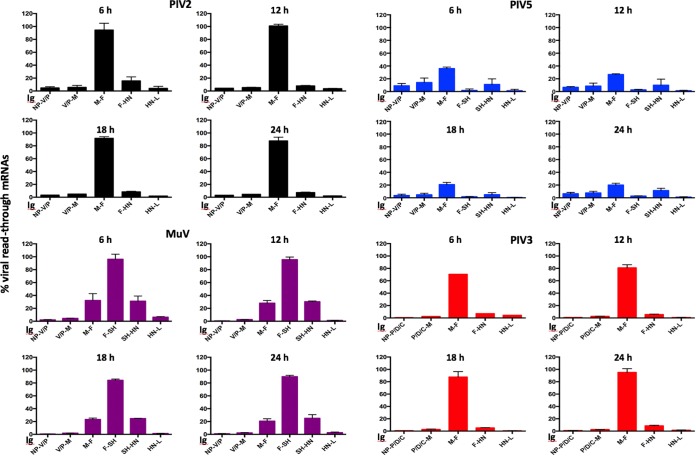
Relative abundance of readthrough mRNAs compared to the average coverage of the gene immediately upstream for PIV2-Co, PIV5-W3, MuV-Enders, and PIV3-Wash. The average coverage of reads overlapping the IG was compared to the average coverage read depth of the gene immediately upstream of the IG region. The bars show SD values based on three independent experiments.

The generation of readthrough mRNAs has been proposed as a secondary mechanism by which paramyxoviruses control the level of production of viral proteins because translation of genes beyond the first represented in the mRNA will not occur. Readthrough mRNAs are generated when vRdRP fails to terminate transcription at a GE site and continues transcribing the IG region and a subsequent gene(s) to produce a bicistronic (or polycistronic) mRNA. The generation of readthrough mRNAs was analyzed by calculating the average coverage depth of reads overlapping each IG region and comparing it to the average coverage depth of reads of the gene immediately upstream (Fig. 5). This method cannot, in principle, distinguish readthrough mRNA from antigenomes, but for the reasons discussed above, the proportion of antigenomes compared to the total viral mRNA was assessed as being very low. In addition, the maximal contribution of antigenomes could not exceed the lowest readthrough rate, which occurred sharply at the boundary between the HN and L genes in all four viruses. Moreover, the contribution of antigenomes would not explain any differences in readthrough transcription at the various gene boundaries. This method also cannot distinguish between bi- and polycistronic mRNAs, which have been shown to be generated in PIV5 and MuV (26). The efficiency of readthrough transcription differed greatly among IG regions and among viruses. Thus, a high level of readthrough occurred at the M:F boundary in each case, but the levels differed, being ∼30% for PIV5-W3 and MuV but 90% for PIV3-Wash and PIV2-Co. Readthrough at the F:SH boundary was ∼2% for PIV5-W3, which is in sharp contrast to that for MuV-Enders, in which it was approximately 91%, slightly lower than the estimated 100% reported using Northern blot analysis (27). Similarly, readthrough at the SH:HN boundary was ∼30% for MuV-Enders but ∼10% for PIV5-W3 (PIV2 and PIV3 lack the SH gene). Significantly lower levels of mRNA readthroughs were observed at other gene boundaries for all viruses (Fig. 5).

### Effects of PIV5 strain.

**FIG 6:**
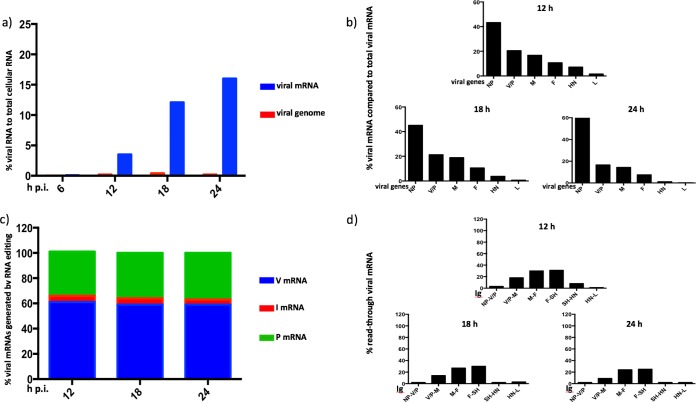
Effects of strain differences on PIV5 transcription and replication. (a) The relative abundance of PIV5-CPI+ mRNA and genome reads were compared to the number of total reads at various times p.i. in A549 cells. Total RNA was isolated and, following physical removal of rRNA and mitochondrial RNA, was subjected to library preparation, HTS, and directional read analysis, followed by bioinformatic removal of residual rRNA and mitochondrial RNA sequences. The mRNA abundance gradient (b), the relative abundance of the P, V, and I mRNAs (c), and the generation of readthrough mRNAs (d) were determined from the data sets as described for Fig. 3 to 5, respectively.

**FIG 7:**
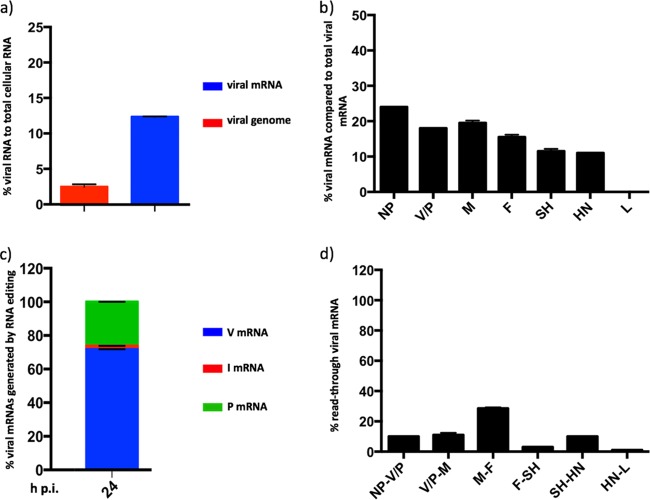
Transcriptional and replicative differences of PIV5 recombinant virus rPIV5-W3:P(F157) (replacement of the serine residue at position 157 by a phenylalanine residue). (a) The relative abundance of rPIV5-W3:P(F157) mRNA and genome reads were compared to the number of total reads at 24 h p.i. A549 cells were infected at an MOI of 10 to 20 PFU/cell and total cell RNA was isolated at various times p.i. rRNA and mitochondrial RNA were physically removed and the RNA was subjected to library preparation, sequencing, and directional analysis, followed by bioinformatic removal of residual rRNA and mitochondrial RNA sequences. The mRNA abundance gradient (b), the relative abundance of the P, V, and I mRNAs (c), and the generation of readthrough mRNAs (d) were determined from the data sets as described for Fig. 3 to 5, respectively.

Single strains of PIV2, PIV3, PIV5, and MuV were used in the analysis described above. To investigate whether strain differences influence the patterns of paramyxovirus transcription and replication, we analyzed the mRNA abundance gradient, RNA editing, and readthrough mRNA profiles of PIV5-CPI+ (Fig. 6). In comparison to PIV5-W3, maximal levels of PIV5-CPI+ transcription were significantly higher at later times (Fig. 6a). Thus, approximately 18% of total RNA at 24 h p.i. was of viral mRNA origin in cells infected with PIV5-CPI+, compared to only 2 to 3% in cells infected with PIV5-W3. This is now known to be because PIV5-W3 [from now where appropriate referred to as PIV5-W3(S157)] transcription is specifically repressed at late times in infection by phosphorylation of a serine residue at position 157 in the P protein (24). Thus, in cells infected with recombinant virus rPIV5-W3:P(F157), in which the serine residue at position 157 in PIV5-W3 was replaced by a phenylalanine residue, approximately 14% of total RNA was of viral origin at 24 h p.i. (Fig. 7a). Similarly, PIV5-CPI+ has a phenylalanine residue at position 157 of the P protein that cannot be phosphorylated. However, initial rates of PIV5-CPI+ transcription were similar to those of PIV5-W3 and significantly lower than those of PIV3-Wash (compare Fig. 3 and 6). However, there were also differences in the mRNA abundance gradient and readthrough mRNA profiles of PIV5-W3(S157) and PIV5-W3(F157) from those of PIV5-CPI+, but not in RNA editing (compare Fig. 3 and 6). In particular, there was a significantly greater dropoff in the abundance of P/V/I mRNAs compared to NP mRNA in cells infected with PIV5-CPI+ compared with those in cells infected with PIV5-W3, and there was greater transcriptional readthrough at the M:SH junction in cells infected with PIV5-CPI+.

## DISCUSSION

Recently there have been several studies that quantified viral mRNAs using HTS for negative-strand viruses such as Ebola, respiratory syncytial, and Hendra viruses (for examples, see references [Bibr B28][Bibr B29][Bibr B32]). For transcriptional studies employing HTS, mRNA from infected cells is typically isolated by poly(A) selection. While directional sequencing of poly(A)-selected RNA and a bioinformatic protocol can be used to separate genome RNA data from mRNA/antigenome data, the method suffers from the disadvantage that high levels of quantifiable genome RNA evidently copurified with the poly(A)-selected mRNA, presumably as a consequence of RNA hybridization. We therefore concluded that directional sequencing of total cell RNA following rRNA (and mitochondrial RNA) reduction was a better approach because it allowed the relative amounts of genome and mRNA/antigenome sequences to be quantified. We have also published recently that sequencing of total RNA following rRNA reduction can be used to detect and quantify defective virus genomes within infected cells without the need for nucleocapsid purification prior to sequencing (24).

Separating mRNA and antigenome data is more problematic because these RNAs are both transcribed from genome templates. However, the contribution of antigenomes to the mRNA/antigenome signal is very small. Thus, the levels of antigenome sequences cannot exceed the contribution of the L mRNA signal, which is very low in comparison with that of other genes. Estimates of antigenome abundance obtained by quantifying sequence reads of the region upstream of the GS site for the NP mRNA also strongly suggested that the contribution of antigome reads to the total mRNA/antigenome reads must be very small. However, these latter estimates were only approximate because this region is small and located at the 3′ end of the genome, where coverage depth declines because during library preparation the sequenced fragments are selected to be of a certain minimal size.

There were clear differences in both the kinetics of viral transcription and the mRNA abundance gradients between PIV2-Co, PIV3-Wash, PIV5-W3, PIV5-CPI+, and MuV-Enders. PIV3-Wash replicated the fastest, with mRNAs contributing approximately 10% of total RNA by 6 h p.i. In contrast, the kinetics of PIV2-Co, PIV5-W3, PIV5-CPI+, and MuV-Enders were significantly slower, with viral mRNAs contributing <1% of total RNA at 6 h p.i., suggesting that there may be something fundamentally different between the mode of PIV3 (respirovirus) replication and that of PIV2, PIV5, and MuV (orthorubulavirus) replication. It will be interesting to determine whether this holds for other viruses in these groups.

The maximal amount of PIV5-W3 mRNA in infected cells was significantly lower than that of the other viruses examined. As discussed above, this is because PIV5-W3 transcription and replication are repressed at late times in infection due to phosphorylation of a serine residue at position 157 on the P protein. PIV5 transcription is not repressed following infection with strains of PIV5, including PIV5-CPI+ and rPIV5-W3:P(F157), that have a phenylalanine residue at position 157, and this is reflected in higher levels of viral mRNA at late times p.i. (24). Interestingly, although the relative levels of mRNA between PIV5-W3(S157) and PIV5-W3(F157) differ significantly at late times, the general pattern of their mRNA abundance gradients and the abundance of readthrough mRNAs are similar but differ from those of PIV5-CPI+. Thus, there is a greater decrease in the relative abundance of the P/V/I mRNAs compared to NP for PIV5-CPI+ than for either PIV5-W3(S157) or PIV5-W3(F157). These results suggest that there may be subtle differences in the control of virus transcription and replication of different paramyxovirus strains. It will therefore be of interest to determine whether other strains of PIV2, PIV3, and MuV show profiles similar to those of the strains used in this study and what, if any, are the biological consequences of such differences.

**FIG 8:**
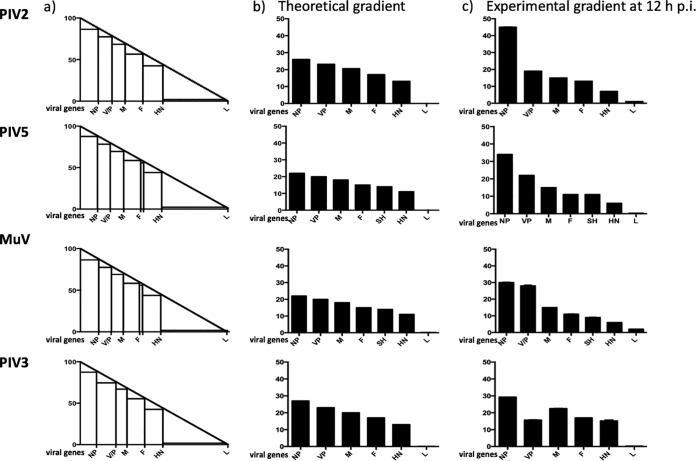
Theoretical mRNA abundance gradients compared to actual gradients in a model in which vRdRP disengages with equal chance at any nucleotide during transcription, and truncated, non-poly(A) mRNAs are rapidly degraded. (a) Model of the relative abundance of individual viral mRNAs in which position 1 of the genome constitutes 100% of transcripts and the last nucleotide constitutes 1 to 2%. The end of each gene is indicated where polyadenylation occurs at the U tract to generate mRNAs that are subsequently translated. In this model it is assumed that transcripts that are prematurely terminated when vRdRP disengages from the genome upstream of the U tract are not polyadenylated and are degraded rapidly. The stepwise transcription profiles therefore reflect the theoretical abundance of polyadenylated mRNAs. (b) The theoretical percentage contribution of polyadenylated viral mRNAs to the total viral mRNA population, as calculated from the theoretical gradient shown in panel a. (c) The mRNA abundance gradient determined experimentally for cells infected with PIV2-Co, PIV5-W3, MuV, or PIV3-Wash at 12 h p.i. as described for Fig. 2.

In the context of the mRNA abundance gradient, PIV3-Wash exhibited a relatively small decline in the relative abundance of the P/D/C, M, F, and HN mRNAs. However, there was a dramatic decrease in the abundance of L mRNA compared to HN mRNA. In comparison, PIV2-Co, PIV5-W3, and PIV5-CPI+ exhibited a relatively large decrease in the relative abundance of P/V mRNA compared to NP mRNA and then a gradual decline until the HN mRNA, before again showing a marked decrease in the abundance of L mRNA. MuV-Enders was similar to PIV2-Co and PIV5-W3, except that the first obvious decrease in abundance occurred between the P/V and M mRNAs. Although the reasons for the decrease in the relative abundance of L mRNA compared to HN mRNA is unclear, it may be that the much greater length of the former is a contributing factor. The generally accepted model for the stepwise reduction in mRNA abundance across the genome is that the vRdRP may disengage from the genome at a GE site, rather than continuing to transcribe downstream genes, but if it does so it must reinitiate at the Le promoter to continue transcribing. An alternative explanation is that vRdRP can disengage at any nucleotide with equal probability, with the aborted, non-poly(A) RNAs being very rapidly degraded (33, 34). Such a scenario would also lead to an apparently stepwise mRNA abundance gradient. To determine whether this model fits the experimental data, a theoretical model of the abundance of viral mRNAs was generated by assuming 100% abundance at position 1 gradually decreasing to 1 to 2% at the last position of the genome (the percentage abundance of L mRNAs) to produce a theoretical mRNA abundance gradient line (Fig. 8a). The intersection of the poly(U) tract with the theoretical transcription line was then used to obtain the theoretical abundance of polyadenylated mRNAs. Interestingly, at 12 h p.i. (a time chosen to minimize any effects of differences in viral mRNA stability but at which appreciable levels of transcription had occurred), PIV3-Wash showed an experimental mRNA abundance gradient that is most similar to the theoretical model. Indeed, the relative abundance of the viral mRNAs, apart from L mRNA, was <1.8-fold different from the relative abundance of the mRNA of the gene immedately upstream. In contrast, L mRNA was >50-fold less abundant than HN. PIV3 is a respirovirus with conserved GS and IG regions, and although differences in the GE sequences and other sequences present in the genome may influence the rates of termination and reinitiation at gene boundaries, it would be surprising if the marked decrease in L mRNA could be explained by the vRdRP disengaging with much greater frequency at the HN-L gene junction than at other gene boundaries. However, further experimental investigations will be needed to determine which of these two models is correct. For PIV2-Co, PIV5-W3, and MuV-Enders (rubulaviruses), the theoretical transcriptional profiles differed significantly from the experimental data for genes near the 3′ promoter. Thus, for PIV2-Co, the amount of V/P mRNA was significantly smaller than that of NP mRNA, whereas for MuV-Enders, the equivalent step decrease in abundance was located between the V/P and M genes. Thereafter, the relative reduction in abundance of viral mRNAs fitted the theoretical model relatively well. Since the intergenic regions of orthorubulaviruses are not conserved within the genome, this suggests that relative mRNA abundance may be determined both by specific disengagement of vRdRP at gene junctions, as has previously been suggested, and by degradation of non-poly(A) mRNAs generated as vRdRP randomly disengages from the template. However, if so, the biological consequences for orthorubulaviruses controlling mRNA abundance in this relatively more complicated manner than PIV3 are not known.

Because eukaryotic ribosomes do not generally recognize internal AUG initiation sites, viral protein expression can be further controlled by the generation of readthrough mRNAs, as downstream genes transcribed as polycistronic mRNAs would not be translated. In agreement with published work (35–37), PIV5-W3, PIV2-Co, and PIV3-Wash displayed a greater degree of readthrough at the M:F junction than other junctions. For PIV5-W3 and MuV-Enders, approximately one-third of transcripts starting from the M gene read into the F gene, whereas PIV3-Wash and PIV2-Co displayed a much higher proportion (approximately 90% to 98%, respectively) of readthroughs, thereby significantly reducing the amount of F synthesized. It has been suggested that such a mechanism may have evolved in order to decrease amount of F made and thus to reduce the cytopathic effects of infection while maintaining the abundance of downstream mRNAs (17, 38). Our results showing that the rate of readthrough of PIV5-W3 at M:F is approximately 3-fold higher than for the other IGs agrees with those of Rassa and Parks (17), who used Northern blot analysis to investigated mRNA readthrough at each gene junction. They did, however, observe a slight change in the rate of readthrough of the M:F gene over time which was not observed during this study. As well as virus factors, host cell differences can also influence the generation of polycistronic mRNAs (39) and may therefore explain the differences between our results and those of Spriggs and Collins (40), who, using Northern blot analysis, showed that approximately equal amounts of F monocistronic and M:F readthrough mRNAs were made during infection with PIV3-Wash. For MuV-Enders, we also show here that readthrough at the F:SH junction at 12 h p.i. was >90%. In agreement, Takeuchi et al. (27) showed that no monocistronic SH or SH-HN bicistronic mRNA was produced by MuV-Enders, although monocistronic HN and SH were made by other strains. However, although in our analysis we detected readthrough sequences between the SH and HN genes, as we cannot distinguish between bicistronic or any other polycistronic mRNAs, it is possible that the SH-HN reads we detected may have arisen from F-SH-HN tricistronic mRNA, which were detected in high abundance by Takeuchi et al. (27).

To initiate RNA synthesis at the Le promoter, the vRdRP recognizes a conserved sequence at the 3′ end of the genome and a set of tandem repeats in the untranslated region of the NP gene that must be in the correct hexamer phase (reviewed in reference 41). This suggests that vRdRP functionality may be controlled by sequence recognition, hexamer phasing, or both. The sequence and hexamer phasing of the GE and GS sites and the IG region in PIV2-Co, MuV-Enders, PIV3-Wash, and both PIV5-W3 and PIV5-CPI+ were analyzed for clues suggesting a mechanism for controlling vRdRP function at the gene junction. For PIV2 there were no obvious differences in the NP GE or the V/P GS that could account for the significant decrease in the abundance of V/P/I mRNA compared to that of NP mRNA. Similarly, no differences in the V/P GE or the M GS could be identified as a possible control mechanism in MuV for the significant decrease in M mRNA abundance compared to V/P/I mRNA abundance. However, there was an A to U change in the GE of the NP gene of PIV5-W3 compared to PIV5-CPI+ that might account for the relatively greater drop in abundance of V/P mRNA to NP mRNA observed in PIV5-CPI+. With regards mutations that may influence the abundance of PIV5 readthrough mRNAs, it has previously been reported that mutations at position 5 in the M GE sequence, can affect the relative abundance of M:F readthrough mRNAs (18). Interestingly, the M GE sequences are identical between PIV5-W3 and PIV5-CPI+, and they have similar levels of M:F readthrough mRNA. However, there are four nucleotide differences at the F GE between PIV5-W3 and PIV5-CPI+, including at position 5, that may explain the higher levels of F:SH readthrough mRNA in PIV5-CPI+.

There were also clear differences between PIV2-Co, PIV3-Wash, PIV5-W3, and MuV-Enders with regard to the relative abundance of the P/V/I/D mRNAs produced by insertion of nontemplated G residues at the editing site. For PIV2-Co and PIV5-W3, the ratios of V to P mRNAs were 3:1 and 2:1, respectively, and together they accounted for more than 94% of all transcripts generated from the P/V gene. This is in contrast to the results of Thomas et al. (42), who found that PIV5 inserted G’s at a ratio of 1:1. The ratio of the V to P mRNAs for MuV-Enders was roughly 1:1, with I mRNAs contributing approximately 5% of mRNAs generated from the P/V/I gene. In PIV3-Wash, the ratio of the P to V to D mRNAs was approximately 3:1:2. The high levels of the PIV3-Wash D and V mRNA produced is surprising given that no biological function has been assigned to the encoded proteins. Although an ancestral ORF is present in the V mRNA, there are two stop codons downstream of the editing site that would result in the production of a truncated V protein that would be highly unlikely to act as an IFN antagonism, as it does in PIV5. However, structural and biochemical analyses have demonstrated that the N-terminally common domain of P and V in PIV5, Sendai virus, and measles virus contain binding sites for NP (7, 43–46), and thus it is possible that PIV3 V and D have roles in maintaining the solubility of NP^0^ soluble prior to encapsidation of the viral genome or antigenome, as has been suggested for PIV5 (7). Alternatively, the V protein of PIV3 may have a role in controlling viral transcription and replication, as has been demonstrated for a number of paramyxoviruses.

## MATERIALS AND METHODS

### Infections.

Human skin fibroblasts (HSFs) and A549 cells (of human adenocarcinomic alveolar basal epithelial origin) were maintained as monolayers in 25-cm^2^ tissue culture flasks (Greiner) in Dulbecco’s modified Eagle’s medium (Invitrogen) supplemented with 10% (vol/vol) heat-inactivated fetal bovine serum (Biowest) and incubated in 5% (vol/vol) CO_2_ at 37°C. The viruses used were PIV2 strain Colindale (PIV2-Co), PIV3 strain Washington/47885/57 (PIV3-Wash [47]), PIV5 strain W3 (PIV5-W3 [48]), MuV strain Enders (MuV-Enders [49]), PIV5 strain CPI+ (PIV5-CPI+ [50]), and PIV5 strain rPIV5-W3:P(F157) (24). Cell monolayers were infected with virus diluted in medium at a multiplicity of infection (MOI) of 10 to 20 PFU per cell, unless stated otherwise. The infected monolayers were placed on a rocker for 1 h to allow adsorption of the virus, after which the inoculum was removed and replaced with medium supplemented with 10% (vol/vol) heat-inactivated fetal bovine serum and incubated in 5% (vol/vol) CO_2_ at 37°C until harvested.

### DNA sequencing.

Cells were scraped into the medium and transferred into 15-ml tubes which were centrifuged at 4,700 rpm for 5 min. The pellet was resuspended in 1 ml of TRIzol (Invitrogen), and an equal volume of ethanol was added. RNA was isolated using a Direct-zol RNA miniprep kit (Zymo) and sequenced directionally, either by selection of poly(A) mRNA using a TruSeq stranded mRNA library preparation kit LS (Illumina) or by reduction of rRNA or rRNA plus mitochondrial RNA using a TruSeq stranded total RNA library preparation kit with a Ribo-Zero human/mouse/rat kit (Illumina) or a Ribo-Zero Gold kit LS (Illumina), respectively. Identical steps for library preparation were then followed (for a full description, see https://support.illumina.com). Quality control and quantification of DNA libraries were monitored using a 2100 Bioanalyzer with 1,000 or 5,000 DNA-specific chips (Agilent) and a Qubit fluorometer (Invitrogen). The libraries were normalized to 10 nM, pooled in equal volumes, and subjected to HTS on an MiSeq or NextSeq instruments (Illumina) to produce paired-end reads in two files (R1 and R2) that contained data obtained with the forward and reverse primers.

### Bioinformatic analyses.

The sequencing data were demultiplexed, and the reads were trimmed to remove adapter sequences and filtered to remove low-quality reads using TrimGalore (available at https://github.com/FelixKrueger/TrimGalore). Read quality (Q score) was restricted to >30.

A bioinformatic pipeline was developed for analyzing viral transcription and replication. The reads contained in the R1 and R2 files were mapped independently to the appropriate reference genome sequence using BWA version 0.7.5a-r405 (51). The reference genomes for PIV2-Co, PIV5-W3, PIV5-CPI+, and MuV-Enders were obtained from GenBank (accession no. AF533012, JQ743318, JQ743321, and GU980052, respectively). The PIV3-Wash sequence was obtained by *de novo* assembly of the read data. The aligned reads were then binned from the R1 and R2 assemblies on the basis of their orientation in relation to the genome sequence and combined to produce two files exclusively containing genome or mRNA/antigenome reads. The reads in these files were then mapped independently to the reference sequence using BWA. The number of reads mapping to the genome and their coverage depth across the genome were ascertained by visualizing these alignments using Tablet version 1.15.09.01 (52). In later stages of the study, the abundances of genome and mRNA/antigenome reads were calculated relative to total read numbers (including cellular reads) from which residual rRNA and mitochondrial RNA reads had been removed. The latter reads were identified by aligning the trimmed, filtered data to reference genomes for human 18S, 28S, 5S, and 5.8S rRNA and mitochondrial DNA (accession numbers NR_003286, NR_003287, X51545, J01866, and NC_012920, respectively).

Relative mRNA abundances were calculated from values for fragments per kilobase of transcript per million mapped reads (FPKM) obtained using RSEM version 1.3.0 (53). FPKM values normalize the abundance of transcripts generated from individual genes to account for differences in gene length, thus allowing the relative amounts of viral mRNA generated from different genes to be compared. However, this method cannot distinguish between alternative transcripts generated by RNA editing. Instead, reads overlapping the RNA editing site were quantified by identifying those containing the 10-nt sequences immediately upstream and downstream of the poly(G) tract in which editing occurs. The numbers of these reads containing 1, 2, 3, 4, 5, 6, or 7 additional G residues were binned individually and compared to the total.

To quantify reads that cross IG regions, the average coverage depth of reads that align to specific genes or that cross the IG region was calculated using SAM2CONSENSUS version 2.0 (available at https://github.com/vbsreenu/Sam2Consensus). The proportion of readthrough mRNAs was calculated by comparing the number of reads that cross the IG region to the average coverage depth of the gene immediately upstream.
